# Elongation Factor 1A-1 Is a Mediator of Hepatocyte Lipotoxicity Partly through Its Canonical Function in Protein Synthesis

**DOI:** 10.1371/journal.pone.0131269

**Published:** 2015-06-23

**Authors:** Alexandra M. Stoianov, Debra L. Robson, Alexandra M. Hetherington, Cynthia G. Sawyez, Nica M. Borradaile

**Affiliations:** 1 Department of Physiology and Pharmacology, Schulich School of Medicine and Dentistry, Western University, London, ON, Canada, N6A 5C1; 2 Department of Medicine, Western University, London, ON, Canada, N6A 5C1; 3 Robarts Research Institute, Western University, London, ON, Canada, N6A 5C1; University of Basque Country, SPAIN

## Abstract

Elongation factor 1A-1 (eEF1A-1) has non-canonical functions in regulation of the actin cytoskeleton and apoptosis. It was previously identified through a promoter-trap screen as a mediator of fatty acid-induced cell death (lipotoxicity), and was found to participate in this process downstream of ER stress. Since ER stress is implicated in the pathogenesis of nonalcoholic fatty liver disease (NAFLD), we investigated the mechanism of action of eEF1A-1 in hepatocyte lipotoxicity. HepG2 cells were exposed to excess fatty acids, followed by assessments of ER stress, subcellular localization of eEF1A-1, and cell death. A specific inhibitor of eEF1A-1 elongation activity, didemnin B, was used to determine whether its function in protein synthesis is involved in lipotoxicity. Within 6 h, eEF1A-1 protein was modestly induced by high palmitate, and partially re-localized from its predominant location at the ER to polymerized actin at the cell periphery. This early induction and subcellular redistribution of eEF1A-1 coincided with the onset of ER stress, and was later followed by cell death. Didemnin B did not prevent the initiation of ER stress by high palmitate, as indicated by eIF2α phosphorylation. However, consistent with sustained inhibition of eEF1A-1-dependent elongation activity, didemnin B prevented the recovery of protein synthesis and increase in GRP78 protein that are normally associated with later phases of the response to ongoing ER stress. This resulted in decreased palmitate-induced cell death. Our data implicate eEF1A-1, and its function in protein synthesis, in hepatocyte lipotoxicity.

## Introduction

Eukaryotic elongation factor 1A-1 (eEF1A-1), the mediator of GTP-dependent recruitment of aa-tRNA to the ribosome, has been shown to participate in diverse cellular processes, including actin bundling and regulation of cell morphology, and cell death [1]. eEF1A-1 is rapidly, post-transcriptionally induced in response to oxidative and endoplasmic reticulum (ER) stress, and promotes cell death under these conditions [2, 3]. It was previously identified as a factor involved in lipotoxicity through a retroviral promoter-trap screen for CHO cell resistance to palmitate-induced cell death [3]. In the same study, it was found to regulate the amount of polymerized actin formed in response to palmitate, preceding cell death [3]. Interestingly, recent studies in yeast suggest a link between the actin bundling and protein translation functions of EF1A that mediates feedback regulation of translation at the level of eIF2α phosphorylation [4]. In mammalian cells, eIF2α phosphorylation and subsequent inhibition of global protein translation, is part of the initial phase of the unfolded protein response (UPR) to ER stress [5]. Thus it is possible that eEF1A-1 could respond to fatty acid-induced ER stress and promote subsequent cell death by regulating both protein synthesis and actin cytoskeleton dynamics.

Nonalcoholic fatty liver disease (NAFLD) encompasses a range of disorders associated with lipid accumulation in hepatocytes, from benign steatosis to nonalcoholic steatohepatitis (NASH) [6, 7]. Although the mechanisms of progression of NAFLD are incompletely understood [7], NASH is often regarded as a consequence of hepatocyte lipotoxicity, which occurs as fatty acid flux to the liver exceeds its ability to oxidize them, to export them into plasma, or to store them safely as triglycerides [8, 9]. Hepatocyte injury and death ensue, in association with hepatic inflammation and the onset of fibrosis [10]. Studies of hepatoma and hepatocyte cultures have revealed that, like many other cell types [11], exposure to excess palmitate (saturated fatty acid) triggers cellular stress responses leading to cell death [12–19]. In contrast, oleate (unsaturated fatty acid) does not cause sufficient cell stress to induce cell death in cultured hepatocytes [16, 20–26]. For *in vitro* studies of lipotoxicity, cells are typically incubated with palmitate (0.5 to 1.0 mM) conjugated to bovine serum albumin (BSA). Palmitate is the most abundant saturated fatty acid in our diet and these concentrations reflect serum values, derived from both free and esterified sources, likely to be observed in obese and metabolic syndrome individuals [27]. In cultured hepatocytes, palmitate overload induces oxidative stress [13–15] and ER stress [12, 18, 19, 28, 29], as indicated by increased cellular accumulation of reactive oxygen species (ROS) and activation of the UPR, respectively. These stressors can lead to cell death via mitochondrial pathways [12–14, 16, 17, 19]. Corresponding observations of hepatocyte oxidative stress, hepatocyte ER stress, and hepatocyte death have been made in several rodent models of NALFD [13–15, 30–32], including leptin-deficient *ob/ob* mice [33–35]. Alterations in hepatic lipid composition during NAFLD suggest that, as in cultured hepatocytes, saturated fatty acids are primarily responsible for lipotoxicity *in vivo* [15, 30, 36].

Here we report for the first time that eEF1A-1 was rapidly, but modestly, induced in HepG2 cells in response to high palmitate and, within hours, partially re-localized from its predominant subcellular location at the ER to newly polymerized actin at the cell periphery, preceding cell death. Specific chemical inhibition of the peptide elongation function of eEF1A-1, using the marine-derived depsipeptide didemnin B, did not prevent the initiation of ER stress by high palmitate. But didmenin B did prevent the recovery of protein synthesis that is known to occur during prolonged ER stress, resulting in decreased palmitate-induced cell death. We further found that liver eEF1A-1 protein was induced during severe hepatic steatosis and ER stress in obese *ob/ob* mice. Based on these data, it is possible that eEF1A-1 is a factor that is induced early and drives the progression of NAFLD by promoting hepatocyte lipotoxicity, in part through its function in protein synthesis at the ER.

## Materials and Methods

### Cell Culture, Treatments, and Transfections

HepG2 cells (ATCC) were grown under standard culture conditions for this cell type. For fatty acid treatments, growth medium was supplemented with palmitate, oleate or a combination of palmitate and oleate (2:3 ratio). Fatty acids were complexed to fatty acid free BSA as previously described [3]. Fatty acid free BSA-supplemented medium was used for control conditions. Fatty acids and BSA were from Sigma. Didemnin B was obtained from the Drug Synthesis and Chemistry Branch, Developmental Therapeutics Program, Division of Cancer Treatment and Diagnosis at the National Cancer Institute, and was dissolved in DMSO.

Human *EEF1A1* cDNA sequence was used to design siRNA template oligonucleotides (Ambion siRNA Target Finder). Two hairpin sequences predicted to reduce eEF1A-1 expression were cloned into pSilencer 2.1-U6 expression vectors (Ambion) that were subsequently transfected into HepG2 cells (X-tremeGENE 9, Roche). Control shRNA expressing cells were generated using a scrambled construct encoding no known target (Ambion). Transfected cells were selected by growth in 500 μg/ml hygromycin (Sigma) for 7 days, which was the minimum dose and time of exposure resulting in cell death of the entire population of untransfected control cells. Initial immunoblot analyses indicated that shRNA against our two chosen target sequences, 5’-AAGTCTGTAATGAAGTGTTAT-3’ and 5’-AAGAGATATGAGGAAATTGTT-3’, decreased eEF1A-1 expression. However, only cells expressing shRNA against the first sequence, which resulted in a 24% reduction in eEF1A-1 protein, survived long enough for further experimentation.

### Immunoblotting

Whole cell and tissue lysates were prepared using RIPA buffer containing protease and phosphatase inhibitors. Ten to 50 μg of lysates were resolved under reducing conditions by 10% SDS-PAGE and transferred to nitrocellulose. eEF1A-1 was detected with a mouse monoclonal antibody (Millipore, Cell Signaling Technology). GRP78, phospho-eIF2α, eIF2α, and actin were detected with rabbit polyclonal antibodies (Cell Signaling Technology, Sigma). Secondary antibodies were HRP-conjugated (Santa Cruz Biotechnology). Bands were visualized by chemiluminescence, and those corresponding to eEF1A-1, GRP78, eIF2α and actin consistently appeared at 50 kD, 78 kD, 38 kD and 42 kD, respectively. Band intensities were quantified using Quantity One (Biorad).

### Cell Death

Following 48 h incubations, cells were stained with annexin V (AnnV) and propidium iodide (PI) (Molecular Probes) and analyzed by flow cytometry (FACS Calibur). Apoptotic cells were AnnV positive and PI negative, indicating intact plasma membranes. Dead cells were AnnV positive and PI positive plus PI positive alone, indicating compromised plasma membrane integrity. Live cells were negative for both AnnV and PI.

### Immunofluorescence Confocal Microscopy

HepG2 cells were plated on coverslips and incubated for 6 h with fatty acids. Cells were fixed with 4% paraformaldehyde, permeabilized with 0.2% Triton X-100, and blocked with 0.2% BSA/10% horse serum in PBS. Coverslips were incubated with eEF1A-1 monoclonal antibody (Millipore), followed by FITC-conjugated secondary antibody (Vector Laboratories). For visualization of ER, cells were incubated with anti-calnexin monoclonal antibody (Cell Signaling Technology) followed by secondary antibody conjugated to Alexa Fluor 546 (Molecular Probes). To visualize lipid droplets, cells were stained with Oil Red O (0.3% Oil Red O in isopropanol and PBS). To visualize polymerized actin (F-actin), cells were stained with rhodamine phalloidin (Molecular Probes). All coverslips were affixed to glass slides with mounting medium containing DAPI (Molecular Probes). Images were generated by confocal laser scanning microscopy (Zeiss LSM 510 Meta Confocal Microscope, London Regional Cell and In Vitro Molecular Imaging Facility). Co-localization was quantified in Image J using Pearson’s correlation coefficient (Rr) where 0 indicates random distribution and 1.0 indicates complete positive correlation between fluorescent signals.

### Subcellular Fractionation

Cells were harvested in 250 mM sucrose and 10 mM Tris HCl containing protease inhibitors, homogenized, and fractions were isolated by sequential centrifugation [37]. Post-mitochondrial supernatants were layered over a 1.3 M sucrose cushion and centrifuged to yield three fractions: a supernatant comprising cytosol, a fraction at the interphase of the sucrose cushion comprising smooth microsomes and Golgi apparatus, and a pellet comprising rough microsomes. For immunoblotting, 25 μg samples of each isolated subcellular fraction were resolved by 10% SDS-PAGE.

### [^3^H] Leucine Incorporation Assay

HepG2 cells were treated as indicated with compounds or DMSO (vehicle control), in the absence or presence of BSA or palmitate. [^3^H]leucine was added to media at a specific activity of 500 kBq/ml for 1 h. Cells and culture media were precipitated with cold 1 M trichloroacetic acid. Precipitates were washed with ethanol, and pellets were solubilized in NaOH prior to scintillation counting. Counts were normalized to total protein.

### MTT Assay

HepG2 cells were treated as indicated for 48 h, followed by incubation for 3 h with 0.5 mg/ml MTT (Sigma) in PBS. Cells and formazan reaction product were solubilized with extraction buffer containing 50% dimethylformamide and 20% SDS prior to reading absorbance at 570 nm.

### Mouse Studies

Six week old male C57BL/6J mice and leptin-deficient (*ob/ob*) mice (Jackson Laboratory) were maintained on AIN-76A semi-purified diet (Harlan Teklad) for 4 weeks. All studies were approved by the Western University Council on Animal Care. Plasma lipids were determined by enzymatic assays (Roche Diagnostics). Blood glucose was determined by glucometer (Bayer Healthcare). Plasma insulin was measured by ultrasensitive ELISA (Alpco Diagnostics). Total liver lipids were extracted by the Folch method and quantified by enzymatic assays (Wako Diagnostics). Protein expression in liver tissue homogenates was determined by immunoblotting, as described earlier. Hepatic tissue lipid droplets and tissue morphology were assessed by light microscopy of Oil Red O and H&E stained frozen tissue sections.

### Statistics

Analyses were performed in GraphPad Prism using Student’s t-test, or ANOVA followed by post hoc tests, as appropriate.

## Results

### HepG2 Cell eEF1A-1 Protein Is Modestly Increased in Response to Palmitate-Induced ER Stress

**Fig 1:**
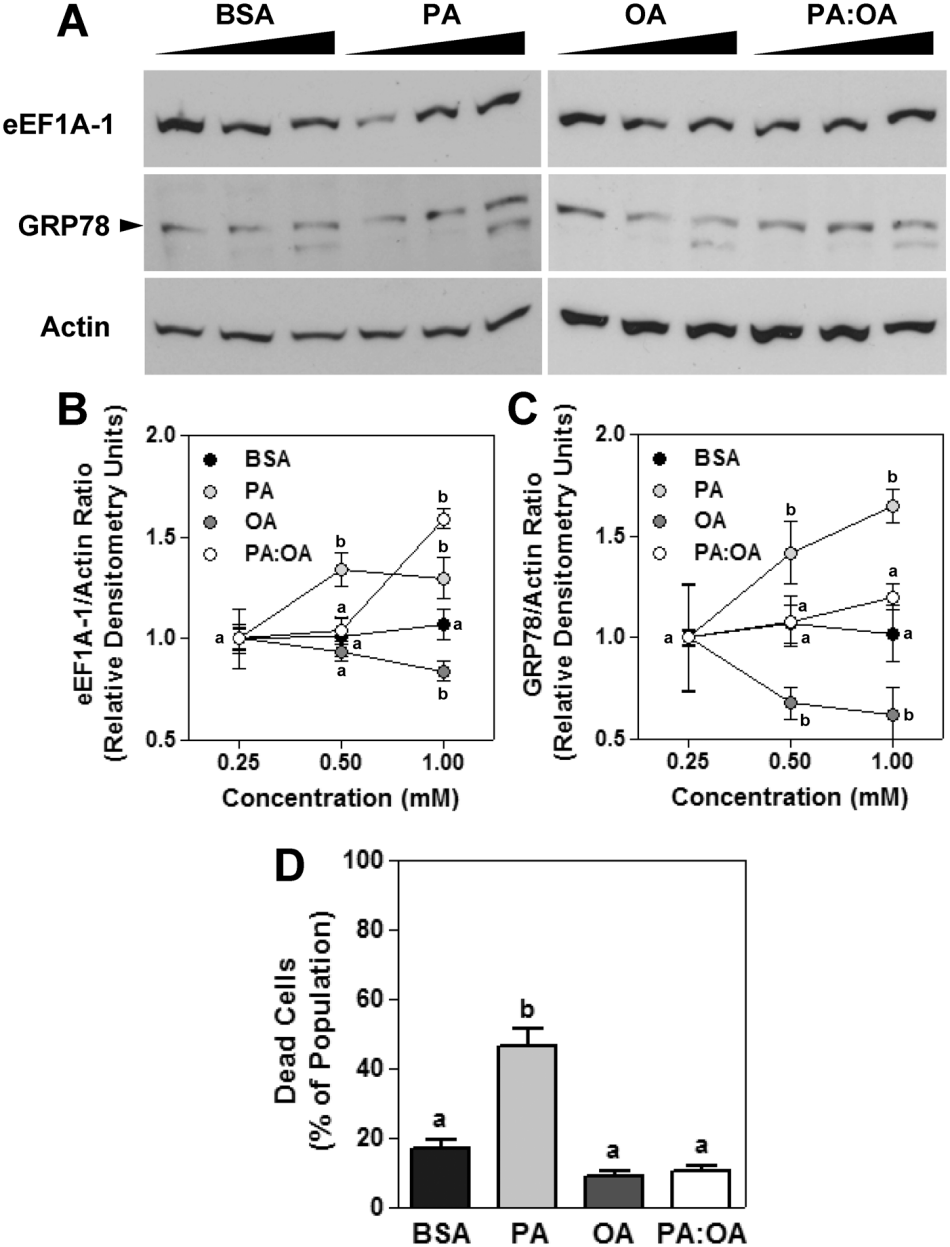
eEF1A-1 Protein is Modestly Increased in HepG2 Cells During Fatty Acid-Induced ER stress, Preceding Cell Death. (A) HepG2 cells were incubated for 6 h with growth media containing increasing concentrations of BSA alone, palmitate (PA), oleate (OA) or a combination of palmitate and oleate (2:3 ratio, PA:OA). All fatty acids were conjugated to fatty acid free BSA at a molar ratio of 2:1. eEF1A-1, GRP78, and actin proteins were detected in whole cell lysates by immunoblotting. Representative blots are shown. (B, C) Immunoblots from experiments performed as described for A were quantified by densitometry. Signals for eEF1A-1 and GRP78 were normalized to actin. (D) HepG2 cells were incubated for 48 h with growth media containing BSA alone, or 1.0 mM fatty acids as in A. Cells were harvested, stained with propidium iodide, and the proportions of dead cells were determined by flow cytometry. All data are means ± SEM for n = 4–8. For B and C different lower case letters are statistically significant at p<0.05 for different concentrations within treatments (BSA or fatty acids).

To characterize the expression of eEF1A-1 in response to lipid overload *in vitro*, we used HepG2 human hepatoma cells because, like intact human and rodent liver, and unlike other hepatocyte lines, they exclusively express the eEF1A-1 variant of eEF1A [38]. In cells incubated for 6 h with growth media containing increasing concentrations of palmitate or palmitate plus oleate, eEF1A-1 protein was increased up to 1.3-fold and 1.6-fold, respectively. In contrast, incubation with oleate modestly decreased eEF1A-1 (Fig 1A and 1B). In palmitate-treated cells, increased eEF1A-1 was accompanied by increased GRP78, indicating the onset of ER stress and the UPR (Fig 1A and 1C). Mean densitometry values for eEF1A-1 normalized to actin (0.25 mM conditions) were 0.93 ± 0.13, 0.82 ± 0.06, 1.08 ± 0.06, and 0.84 ± 0.04 for fatty acid free BSA, palmitate, oleate, and palmitate plus oleate, respectively. Consistent with earlier studies in hepatocytes [12–26, 28], treatment with palmitate for 48 h resulted in a 2.7-fold increase in cell death compared to BSA alone (Fig 1D), while no differences in cell death were observed in cells treated with oleate. The dissociation of increased eEF1A-1 from cell death in cells treated with palmitate plus oleate (Fig 1A and 1B), despite evidence of mild ER stress (Fig 1A and 1C), likely reflects the activation of cytoprotective mechanisms by oleate [20, 39].

### eEF1A-1 Is Partially Re-localized from the ER to Polymerized Actin during Palmitate-Induced Stress

eEF1A-1 has been reported, in proteomic analyses, to associate with lipid droplets and the ER in skeletal myocytes, intestinal enterocytes, and pancreatic β-cells [40–44]. It also binds and remodels the actin cytoskeleton, which undergoes dramatic changes during apoptosis and cell death [1, 45]. However, little is known of the subcellular distribution of eEF1A-1 during cell stress and how this may relate to its role(s) in cellular stress responses, which involve modulation of both protein synthesis and actin cytoskeleton dynamics. To assess the subcellular localization of eEF1A-1 in hepatocytes under basal conditions and during lipid overload, HepG2 cells were incubated with or without fatty acids for 6 h, fixed, immunostained, and imaged by confocal fluorescence microscopy.

**Fig 2:**
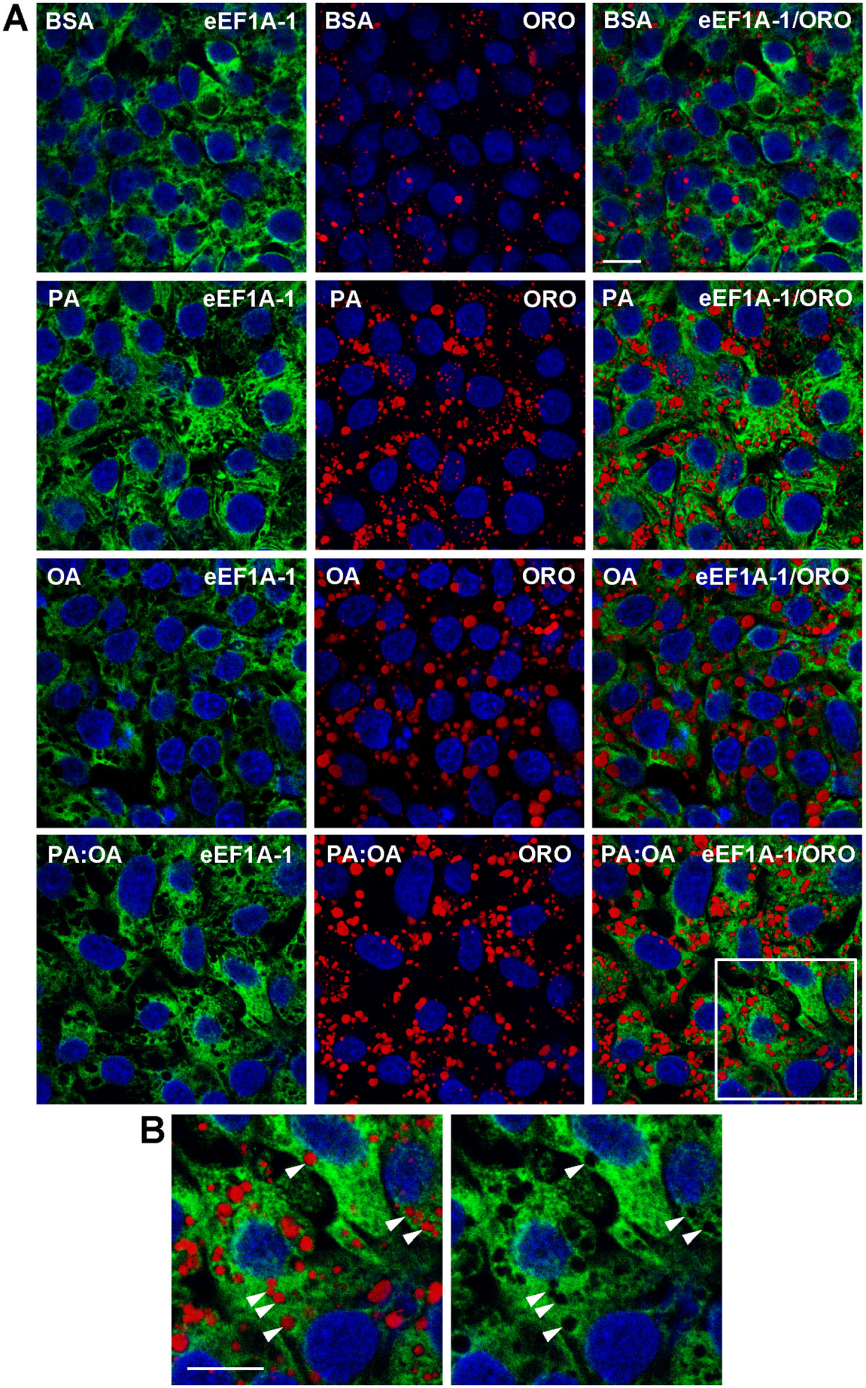
eEF1A-1 Does Not Appear to Co-localize with Lipid Droplets in HepG2 Cells. (A) HepG2 cells were incubated for 6 h with growth media containing BSA alone, or fatty acids as described in Fig 1, at a total concentration of 1.0 mM. eEF1A-1 and lipid droplet localization were assessed by confocal fluorescence microscopy of fixed cells. eEF1A-1 was visualized using anti-eEF1A monoclonal antibody followed by secondary conjugated to FITC (green). Lipid droplets were visualized using Oil Red O (ORO) to stain neutral lipids (red). Nuclei were counterstained with DAPI (blue). (B) Enlargements of the white outlined area in A indicate no co-localization between eEF1A-1 and cytosolic neutral lipid droplets (white arrowheads). Scale bars represent 10 μm. Representative images from n = 3 are shown.

We observed no co-localization of eEF1A-1 with cytosolic neutral lipid droplets under any condition (Fig 2A). Enlargement of the outlined area in Fig 2A indicated that eEF1A-1 surrounds lipid droplets but does not decorate these neutral lipid stores (Fig 2B). However, these images confirmed the modest changes in signal intensity for eEF1A-1 that we observed in response to fatty acids by immunoblotting (Fig 1).

**Fig 3:**
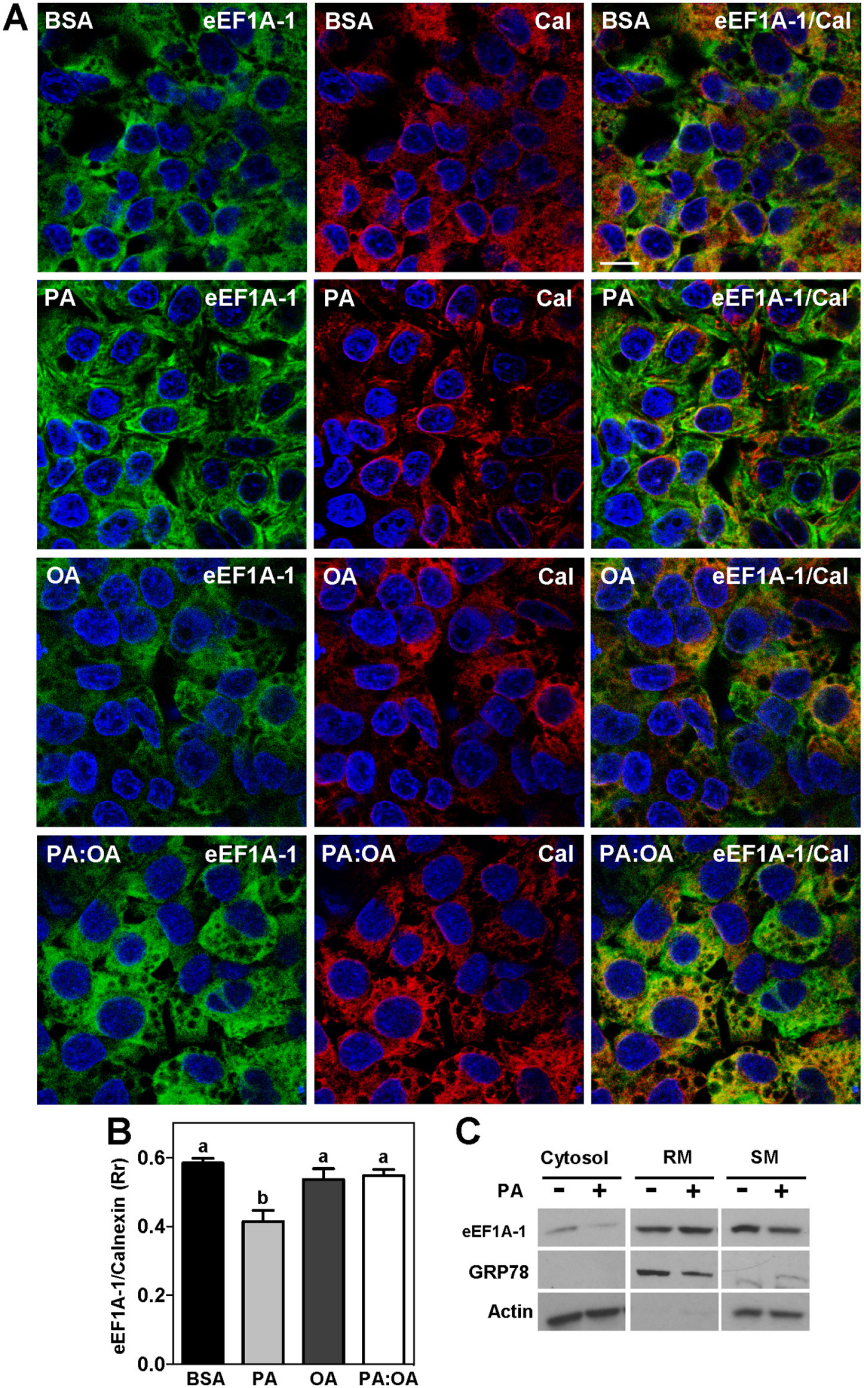
eEF1A-1 Co-localization with the ER is Decreased During Exposure to Excess Palmitate. (A) HepG2 cells were incubated for 6 h with growth media containing BSA alone, or fatty acids as described in Fig 1, at a total concentration of 1.0 mM. eEF1A-1 and ER localization were assessed by confocal fluorescence microscopy of fixed cells. eEF1A-1 was visualized (green) as in Fig 2. The ER membrane protein, calnexin (Cal), was visualized using anti-calnexin monoclonal antibody followed by secondary conjugated to Alexa Fluor 546 (red). Nuclei were counterstained with DAPI (blue). Yellow indicates regions of co-localization between eEF1A-1 and calnexin. Scale bar represents 10 μm. Representative images for n = 3 are shown. (B) Co-localized signal for eEF1A-1 and calnexin in A (yellow) was quantified using Pearson’s correlation coefficient (Rr) to assess overlap between eEFIA-1 (green) and calnexin (red). Data are means ± SEM for n = 3. Different lower case letters are statistically significant at p<0.05. (C) eEF1A-1 protein was detected in cytosol, rough microsomes (RM), and smooth microsomes (SM) isolated by centrifugation from HepG2 cells incubated with or without palmitate, as for A. GRP78 and actin were detected to determine the relative enrichment and contamination of each fraction. Representative blots for n = 3 are shown.

Consistent with studies in other cell types [42–44], we found significant co-localization of eEF1A-1 with the ER membrane protein, calnexin, under basal conditions (Fig 3A, BSA). This co-localization was decreased by 30% in the presence of palmitate (Fig 3B). Subcellular fractionation of HepG2 cells by centrifugation further confirmed that eEF1A-1 is generally enriched in ER fractions (Fig 3C). Although it is tempting to quantitate these data, the procedure of isolating ER fractions from cells undergoing lipotoxicity is not suitable for accurate assessments of changes in protein localization under these conditions. We and others have previously shown that incubation with palmitate rapidly induces dramatic changes in ER lipid composition, structure and integrity, including dilatation of the ER due to increased incorporation of palmitate into ER membrane phospholipids [20, 46, 47]. Indirect evidence of these changes can be seen in images of palmitate-treated cells immunostained for calnexin (Fig 3A **)**. These effects confound the isolation and subsequent protein analyses of equivalent quantities of this organelle between control and palmitate-treated cells.

**Fig 4:**
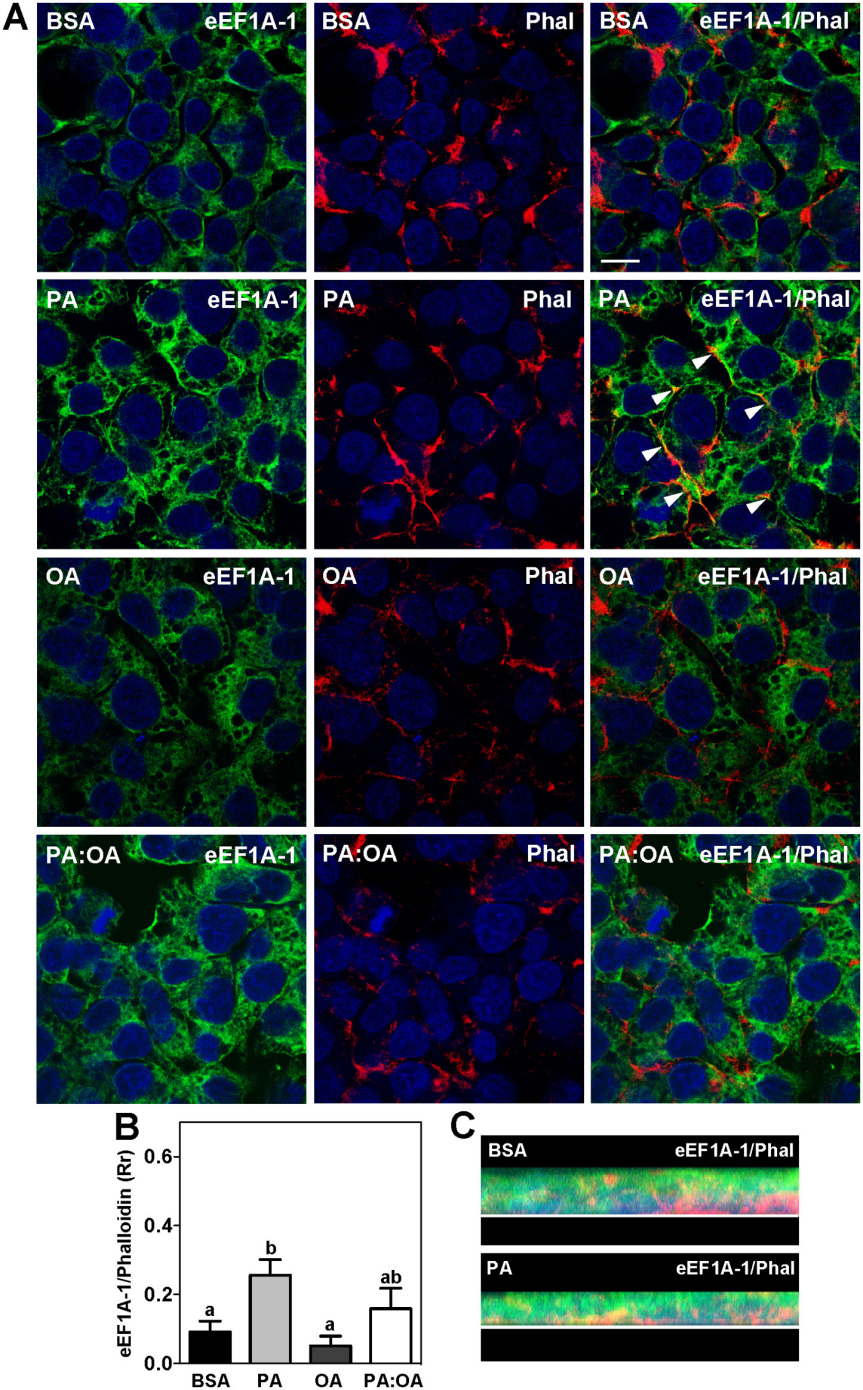
eEF1A-1 Co-localization with the Actin Cytoskeleton is Increased During Exposure to Excess Palmitate. (A) HepG2 cells were incubated for 6 h with growth media containing BSA alone, or fatty acids as described in Fig 1, at a total concentration of 1.0 mM. eEF1A-1 and polymerized actin localization were assessed by confocal fluorescence microscopy of fixed cells. eEF1A-1 was visualized (green) as in Fig 2. F-actin was visualized using rhodamine phalloidin (Phal) (red). Nuclei were counterstained with DAPI (blue). Yellow indicates regions of co-localization between eEF1A-1 and F-actin (white arrowheads). Scale bar represents 10 μm. Representative images from 4 independent experiments are shown. (B) Co-localized signal for eEF1A-1 and F-actin in A (yellow) was quantified using Pearson’s correlation coefficient (Rr) to assess overlap between eEFIA-1 (green) and phalloidin (red). Data are means ± SEM for n = 4. Different lower case letters are statistically significant at p<0.05. (C) Images constructed from a z-series of optical sections of cells from A treated with BSA and PA, respectively. The white line represents the surface to which cells were adhered.

Interestingly, decreased co-localization of eEF1A-1 with the ER in response to excess palmitate was accompanied by increased co-localization (2.8-fold) with the actin cytoskeleton (F-actin) at the cell periphery (Fig 4A and 4B). Previous work has shown that palmitate exposure induces significant actin cytoskeleton remodeling in fibroblasts preceding cell death [3]. This may also occur in HepG2 cells, as cortical actin fibers appeared more pronounced in cells exposed to high palmitate (Fig 4A). Images constructed from a z-series of optical sections showed that, under basal conditions, F-actin was primarily localized to the basolateral surface of the cell monolayer while eEF1A-1 was localized apically (Fig 4C, BSA). Treatment with palmitate resulted in increased co-localized signal at the basolateral surface (Fig 4C).

### Specific Chemical Inhibition of eEF1A-1 Peptide Elongation Function Decreases Palmitate-Induced Cell Death

**Fig 5:**
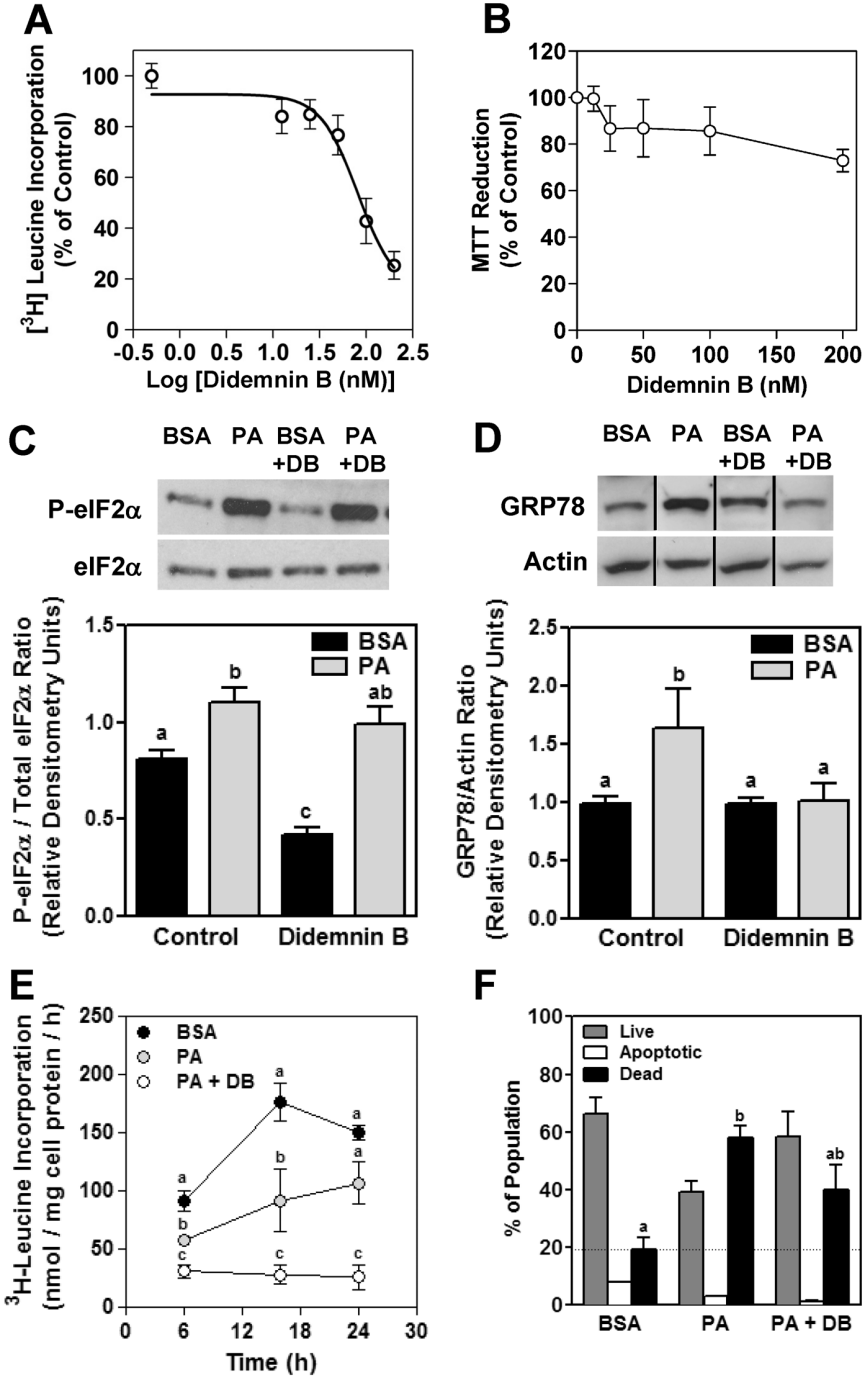
Chemical Inhibition of eEF1A-1 Peptide Elongation Activity Decreases Palmitate-Induced Cell Death. (A) HepG2 cells were treated for 48 h with didemnin B (DB), followed by assessment of total protein synthesis by [^3^H] leucine incorporation. The IC_50_ for protein synthesis was 80 nM. (B) Cells were incubated as in A, followed by assessment of cellular metabolism by MTT reduction. (C, D) HepG2 cells were incubated for 6 h with growth media containing BSA or 1.0 mM palmitate (PA), as described in Fig 1, in the presence or absence of 80 nM didemnin (DB). Phosphorylated and total eIF2α, and GRP78 proteins were detected in whole cell lysates by immunoblotting. Bands were quantified by densitometry and normalized to total eIF2α or actin, as indicated. Representative blots are shown. Black vertical lines on the blots in D indicate that lanes from the same blot were re-ordered for presentation purposes. (E) Cells were incubated for the times indicated with growth media containing BSA or 1.0 mM PA, in the presence or absence of 80 nM DB, followed by assessment of total protein synthesis by [^3^H] leucine incorporation. (F) HepG2 cells were incubated for 48 h with PA, in the presence or absence of 80 nM didemnin (DB). Cells were harvested and stained with PI and AnnV. Percentages of apoptotic and dead cells were determined by flow cytometry. For A and B, data are percentages of control (vehicle) ± SEM for n = 3. For C, D, E, and F, data are means ± SEM for n = 4. Different lower case letters are statistically significant at p < 0.05.

Given the diverse effects of eEF1A-1 on cellular processes, we chose to determine whether the canonical peptide elongation activity of eEF1A-1 plays a role in lipotoxicity. Didemnin B is a cyclic depsipeptide produced by marine tunicates as a chemical defense [48]. It specifically binds GTP-bound eEF1A-1, in a location between the aa-tRNA-binding and GTP-binding domains, but distinct from the actin-binding domain. Didemnin B thereby specifically inhibits eEF1A-1 release from the ribosomal A-site, preventing peptidyl-tRNA translocation and subsequent peptide elongation [49]. To determine the IC_50_ of didmenin B for protein synthesis, cells were treated for 48 h with increasing concentrations of didemnin B, followed by assessment of total protein synthesis by [^3^H]leucine incorporation (Fig 5A). Cell viability, measured by MTT assay, was not affected at the determined IC_50_ concentration of 80 nM (Fig 5B). Subsequently, cells were treated with palmitate, with or without 80 nM didemnin B, followed by assessment of ER stress (6 h), protein synthesis (6–24 h), and cell death (48 h). Didemnin B did not prevent palmitate-induced ER stress and initiation of the UPR, as indicated by increased eIF2α phosphorylation at 6 h (Fig 5C). However, didemnin B did prevent upregulation of GRP78 protein (Fig 5D), in association with sustained inhibition of protein synthesis (Fig 5E). Subsequent palmitate-induced cell death was decreased by approximately 50% at 48 h (Fig 5F). These data suggest that the protein synthesis function of eEF1A-1 promotes lipotoxicity, and that inhibition of this activity mitigates lipotoxic cell death in hepatocytes.

### Modest Long-Term Inhibition of eEF1A-1 Expression Alters HepG2 Cell Morphology

**Fig 6:**
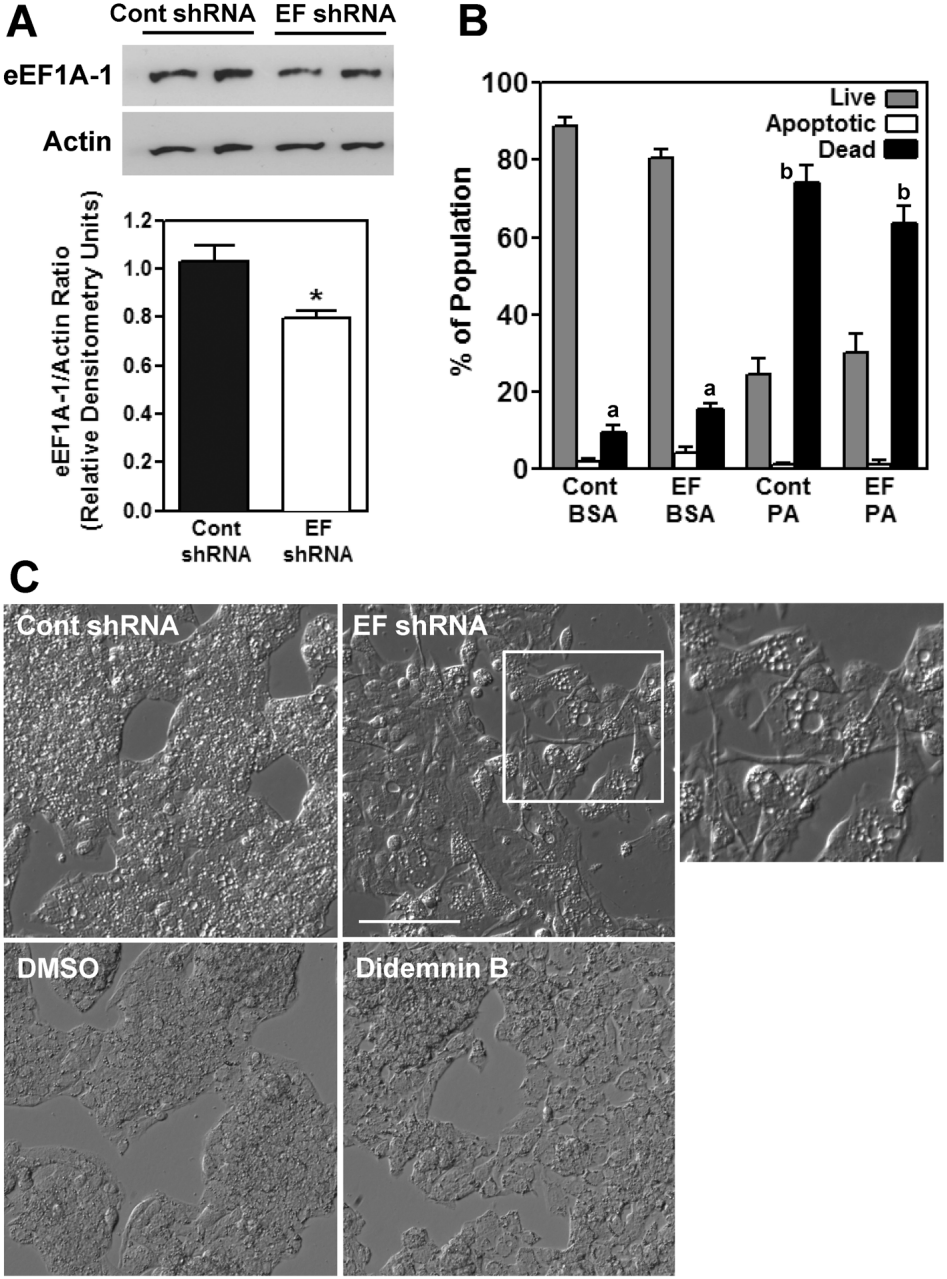
Modest Long-term Inhibition of eEF1A-1 Expression Alters HepG2 Cell Morphology. (A) Stable HepG2 cell populations were generated expressing either control shRNA against no known target (Cont shRNA), or shRNA targeted against *EEF1A1* (EF shRNA). eEF1A-1 and actin protein levels from whole cell lysates were detected by immunoblotting. Representative blots are shown. Bands were quantified by densitometry and normalized to actin. Data are means ± SEM for n = 4 experiments, * p < 0.05. (B) Control shRNA and EF shRNA expressing cells were incubated for 48 h with growth media containing either BSA or 1.0 mM palmitate (PA). Cells were harvested and stained with PI and Ann V. Percentages of apoptotic and dead cells were determined by flow cytometry. Data are means ± SEM for n = 3. (C) Light microscopy of live cells showing morphology of control shRNA or EF shRNA expressing cells and wild type HepG2 cells treated with either DMSO or didemnin B (80 nM) for 48 h. The white outline indicates the enlarged area. Scale bar represents 100 μm.

Knockdown of eEF1A-1 has previously been shown to protect rodent fibroblast cell types (CHO cells and H9c2 rat cardiomyocytes) from palmitate induced cell death [3]. To determine whether a similar effect would occur in human hepatocytes, we opted for a stable shRNA strategy, since our cell death assays required extended incubations which exceeded the window of mRNA silencing achievable with transient siRNA delivery in our system. We generated 3 HepG2 cell populations: two with stable expression of two different shRNA predicted to target eEF1A-1, and one with shRNA expression against no known protein. The population which exhibited the largest decrease in eEF1A-1 expression did not remain viable with sustained eEF1A-1 knockdown, and unfortunately could not be included in further analyses. We were, though, able to sustain growth of a population in which eEF1A-1 protein was decreased by 24% (EF shRNA) compared to the non-protein targeting control (Cont shRNA) (Fig 6A). EF shRNA cells were not significantly resistant to palmitate-induced cell death (Fig 6B). However, these cells exhibited a remarkably altered morphology compared to control cells, including elongated projections extending from an enlarged cell body (Fig 6C). A similar cell shape was observed in the knockdown cell line which did not survive. This morphology was not evident in wild type HepG2 cells treated with the specific inhibitor of eEF1A-1 peptide elongation activity, didemnin B (Fig 6C), suggesting this change in cell shape was not due to inhibition of eEF1A-1 protein synthesis activity alone.

### eEF1A-1 Protein Is Increased in the Livers of Obese Mice with Severe Hepatic Steatosis and ER Stress

**Fig 7:**
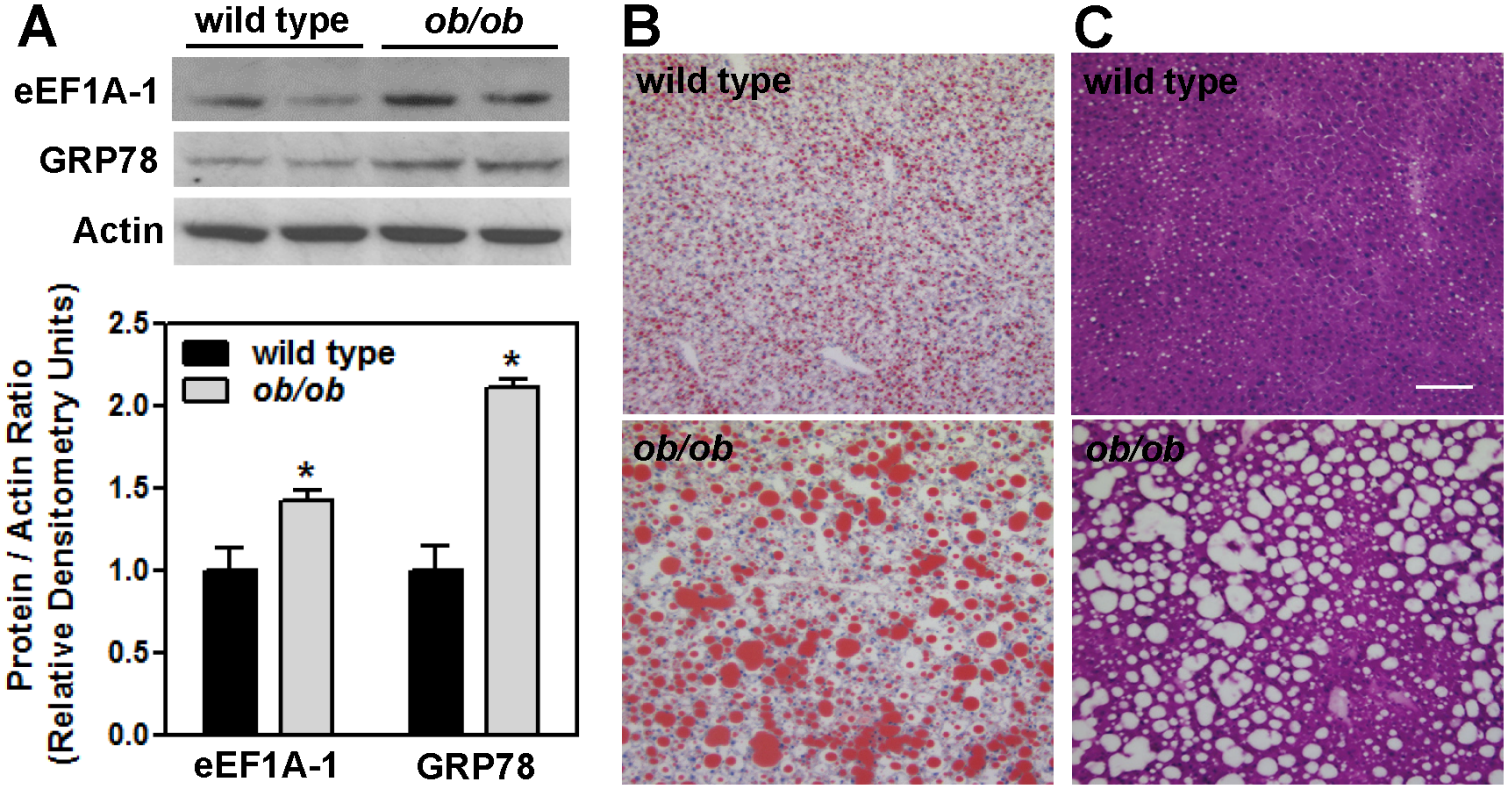
Liver eEF1A-1 Protein is Increased in Obese Mice with Severe Hepatic Steatosis and ER stress. (A) 6 week old male wild type (C57BL/6J) or leptin-deficient (*ob/ob*) mice were maintained on AIN-76A diet for 4 weeks. eEF1A-1, GRP78, and actin proteins were detected by immunoblotting whole tissue homogenates from liver, and quantified by densitometry. Representative blots are shown. Data are means ± SEM for n = 8, * p < 0.05. (B, C) Representative images of Oil Red O and H&E stained frozen liver tissue sections from C57BL/6J (top panels) and *ob/ob* (bottom panels) mice. Scale bar represents 100 μm.

Hepatic eEF1A-1 and GRP78 proteins were significantly increased in obese, leptin deficient *ob/ob* mice that were maintained on AIN-76A diet (12% of calories from fat, 51% of calories from sucrose) for 4 weeks (Fig 7A). Compared to wild type mice, *ob/ob* mice had severe hepatic steatosis (Fig 7B) that was associated with significantly increased liver mass, liver lipids, and characteristics of metabolic syndrome including hyerpinsulinemia, elevated HOMA-IR, and hypercholesterolemia (Table 1). Consistent with previous studies of these mice, we did not observe significant inflammatory infiltrates or fibrosis in *ob/ob* livers (Fig 7C). These data suggest that liver eEF1A-1 is induced during severe hepatic steatosis and the onset of hepatic ER stress.

**Table 1:** Parameters of Metabolic Disease and NAFLD in a Mouse Model of Obesity.

Parameter	C57BL/6J	ob/ob
Body weight (g)	22.7 ± 0.6	44.7 ± 0.8*
Epididymal fat weight (g)	0.43 ± 0.04	3.29 ± 0.09*
Blood glucose (mmol/l)	7.5 ± 0.5	8.4 ± 1.1
Plasma insulin (ng/ml)	0.47 ± 0.22	14.26 ± 1.74*
HOMA-IR	3.8 ± 1.6	135.7 ± 34.1*
Plasma triglycerides (mmol/l)	0.40 ± 0.12	0.30 ± 0.06
Plasma cholesterol (mmol/l)	2.77 ± 0.62	6.38 ± 0.41*
Liver weight (g)	0.96 ± 0.01	3.29 ± 0.09*
Liver triglycerides (mg/g)	25.04 ± 1.58	223.15 ± 35.08*
Liver cholesteryl esters (mg/g)	1.26 ± 0.28	5.72 ± 0.24*
Liver free cholesterol (mg/g)	1.65 ± 0.06	2.31 ± 0.08*

Six week old male C57BL/6J mice and leptin-deficient (*ob/ob*) mice were maintained on semi-purified diet for 4 weeks. Data are means ± SEM, for n = 8.

* p < 0.05 for *ob/ob* mice compared to wild-type C57BL/6J mice.

## Discussion

Despite being the most common chronic liver disorder worldwide, the pathways and factors that mediate progression of NAFLD from benign steatosis to more severe disease are not completely understood [7, 9]. Based on previous characterization of eEF1A-1 as a potential mediator of fatty acid-induced cell death (lipotoxicity), downstream of ER stress [3], we proposed a role for this protein in hepatocyte lipotoxicity and investigated its mechanism of action. Our studies revealed that: 1) during exposure of hepatocytes to high palmitate, eEF1A-1 protein is modestly induced and partially redistributes from its predominant subcellular location at the ER to polymerized actin at the cell periphery, preceding cell death, 2) chemical inhibition of eEF1A-1 peptide elongation activity decreases palmitate-induced death in hepatocytes in association with sustained inhibition of protein synthesis, and 3) liver eEF1A-1 protein is induced in obese mice with severe hepatic steatosis. These observations are consistent with a model in which the majority of eEF1A-1 is localized to the ER in hepatocytes under basal conditions, where it participates in the synthesis of proteins that are processed through this organelle. But, as saturated fatty acid influx exceeds the capacity of the cell to process this substrate, and cell stress ensues, eEF1A-1 is induced and partially displaced from the ER toward the actin cytoskeleton at the cell periphery. Based on previous work, it is possible that eEF1A-1 in this location may contribute to cytoskeletal remodeling that precedes cell death [1, 3, 50], and/or to anoikis signaling [51]. Chemical inhibition of eEF1A-1 peptide elongation activity (using didemnin B) during this process appears to reduce the burden of protein synthesis at the ER, potentially allowing for restoration of ER homeostasis, and the prevention of cell death through this pathway.

In light of proteomic analyses identifying eEF1A-1 in lipid droplet preparations from various metabolic cell types [40, 41, 52], and reports of abundant eEF1A-1 expression in adipose [53] and steatotic tissues [3], it could be proposed that eEF1A-1 regulates lipid droplets in a manner that promotes lipotoxicity. However, our confocal microscopy data show that eEF1A-1 does not directly associate with or decorate lipid droplets, as is typically seen with *bona fide* lipid droplet proteins such as perilipin [54]. The fact that eEF1A-1 is extensively localized to the ER, the site of lipid droplet formation [54], suggests that isolation of lipid droplets for proteomic analyses may result in preparations that include some primarily ER localized proteins, such as eEF1A-1.

The increased co-localization of eEF1A-1 with newly polymerized actin (F-actin) at the cell periphery that we observed in high palmitate, combined with our previous evidence that palmitate increases F-actin stress fibers in fibroblasts prior to cell death [3], suggests that eEF1A-1 participates in lipotoxicity in part by promoting actin polymerization. Localization of eEF1A-1 to F-actin at the cell periphery and basolateral cell surface during palmitate-induced stress may also indicate its accumulation near membranes that are in contact with adjacent cells and the surface of the cell culture vessel. This would be in line with recent evidence implicating eEF1A-1 in anoikis (cell detachment-initiated cell death) through increased localization to the cell membrane, where it acts as a receptor for exposed anti-adhesive sites in fibronectin [51]. Whether anoikis is a predominant mode of cell death in hepatocytes during the progression of NAFLD is an interesting possibility that remains to be determined.

Although the peptide elongation function of eEF1A-1 is well characterized, it has not been previously implicated in lipotoxicity or ER stress responses. Work from several groups indicates that activation of the UPR is, itself, sufficient to cause hepatic steatosis [5, 55]. This suggests a disease scenario in which increased fatty acid flux to the liver initially induces ER stress, which stimulates hepatocyte *de novo* lipogenesis, exacerbating hepatic ER stress and the progression of NAFLD. In this setting, either inhibition of protein synthesis or enhancement of protein folding may decrease the burden on the ER, decreasing subsequent activation of ER stress response and cell death pathways. This concept is supported by recent studies with small molecules such as 4-phenylbutyrate acid and TUDCA, which improve ER stress and liver function in mice and in humans by increasing protein folding capacity and the appropriate trafficking of misfolded proteins [56]. Didemnin B is not a small molecule facilitator of protein folding, but rather is a naturally occurring, marine-derived cyclic depsipeptide that inhibits protein synthesis by specifically inhibiting the peptide elongation activity of eEF1A-1 [49]. In our experiments, didemnin B (at its IC_50_) prevented the recovery of protein synthesis that is known to occur during prolonged ER stress and to contribute to subsequent cell death [57, 58]. This was associated with decreased palmitate-induced cell death. Consistent with recent studies in mutant yeast strains [4], inhibition of eEF1A-1 peptide elongation activity with didemnin B under basal conditions did not trigger feedback stimulation of eIF2α phosphorylation in our system. Instead, it is likely that didemnin B does not prevent the early effects of excess palmitate on ER membrane composition [46] that trigger ER stress and eIF2α phosphorylation; but that its subsequent, sustained inhibition of eEF1A-1-dependent protein synthesis at the ER is protective during exposure to lipotoxic conditions. Recent studies of the mechanism of action of didemnin B suggest that this sustained inhibition of protein translation may be partly mediated through subsequent (indirect) downstream inhibition of mTORC1 [59]. Our observations are consistent with this possibility. Thus our data suggest, for the first time, that the peptide elongation activity of eEF1A-1 is involved in the process of fatty acid-induced cell death.

Inhibition of *EEF1A1* expression, using shRNA, confirmed the multiple functions of this protein in HepG2 cells. The degree of knockdown we achieved (24%) did not confer statistically significant protection against palmitate-induced cell death. This is consistent with previous observations in fibroblast cell types, which suggest that there is a direct correlation between the degree of knockdown of eEF1A-1 protein and resistance to palmitate cytotoxicity [3]. However, these cells exhibited a distinct enlarged and elongated morphology which was not observed upon specific chemical inhibition of eEF1A-1 peptide elongation activity with didemnin B. Yeast with point mutations in the actin binding domain of EF1A (yeast homolog of eEF1A-1) were previously found to be larger in size with pronounced elongated buds, but no defect in protein elongation, highlighting the distinct functions of this factor in translation and in regulation of the actin cytoskeleton [60]. Thus, it is likely that the reduction in total eEF1A-1 protein in our system was sufficient to disrupt eEF1A-1-mediated regulation of the actin cytoskeleton, resulting in altered cytoskeletal organization that impacts cell morphology.

Our data from *ob/ob* mice show that liver eEF1A-1 protein is increased during severe hepatic steatosis, providing some evidence that this factor responds to conditions of hepatic lipotoxicity *in vivo*. This is in agreement with previous findings of increased myocardial eEF1A-1 protein in mice with lipotoxic cardiomyopathy [3]. Induction of eEF1A-1 in these models likely reflects the oxidative and ER stress known to exist under these conditions [11]. In further support of this concept, its induction has been shown in skeletal muscle [61] and in kidney [62] during the oxidative stress of type 1 diabetes. Whether increased hepatocyte eEF1A-1 protein and its subcellular redistribution are markers of NAFLD progression, and whether inhibition of eEF1A-1 activity has therapeutic potential, remain as possibilities for further investigation. Nonetheless, our data point to a role for eEF1A-1 in the promotion of lipotoxicity in hepatocytes, and indicate that inhibition of its elongation activity mitigates fatty acid-induced cell death *in vitro*.
